# Prevalence and predictors of posttraumatic stress disorder and depressive symptoms among burn survivors two years after the 2015 Formosa Fun Coast Water Park explosion in Taiwan

**DOI:** 10.1080/20008198.2018.1512263

**Published:** 2018-09-11

**Authors:** Yi-Jen Su

**Affiliations:** a Graduate Institute of Behavioral Sciences, Chang Gung University, Taoyuan, Taiwan; b Department of Psychiatry, Chang Gung Memorial Hospital, Linkou, Taoyuan, Taiwan

**Keywords:** Burn injury, posttraumatic stress disorder, depression, predictors, cognition, disaster, random forest regression, Lesiones por quemadura, Trastorno de Estrés Postraumático, DepresiónPredictores, CogniciónDesastres, Regresión de bosques aleatoria., 烧伤; 创伤后应激障碍, 抑郁; 预测; 认知; 灾害, 随机森林回归, • PTSD and MDD were prevalent in burn survivors of 2015 Formosa Fun Coast Water Park explosion in Taiwan.• Theory-derived cognitive variables predicted PTSD symptoms post-burn better than risk factors from the meta-analysis and burn-related variables.• Burn severity was uncorrelated with PTSD symptoms post-burn.Negative appraisal of symptoms and maladaptive cognitive coping were the most important predictors of PTSD symptoms after burn.

## Abstract

**Background**: Posttraumatic stress disorder (PTSD) and depressive symptoms are relatively common in burn survivors. Several previously reported risk factors (e.g. burn severity) have not consistently predicted psychological adjustment post-burn. Empirically-derived risk factors of PTSD from the meta-analysis and theory-derived cognitive variables may be of great predictive value.

**Objective**: This study investigated the prevalence of probable DSM-5 PTSD and major depression (MDD) and the predictors of PTSD and depressive symptoms in burn survivors of the 2015 Formosa Fun Coast Water Park explosion. Three sets of predictors were examined: (a) burn-related variables; (b) empirically-derived risk factors from the meta-analysis; and (c) theory-derived cognitive variables.

**Method**: Participants were 116 burn survivors of the Formosa Fun Coast Water Park explosion. The mean age at the disaster was 22.3 ± 4.2 years; the average total body surface area burned (TBSA) was 49.5%.

**Results**: Of our participants, 12.9 and 20.7% met DSM-5 probable PTSD and MDD two years after the Formosa Fun Coast Water Park explosion. No gender differences were observed. For the prediction of PTSD symptoms post-burn, theory-derived cognitive variables (adjusted *R* ^2^ = .562, 95% CI [.423, .638]) performed best and provided significantly better prediction than empirically-derived risk factors from the meta-analysis (adjusted *R* ^2^ = .337, 95% CI [.180, .412]) and burn-related variables (adjusted *R* ^2^ = .313, 95% CI [.156, .389]). In contrast, the three sets of variables examined provided similar predictions for depressive symptoms post-burn (adjusted *R* ^2^ *= *.267–.295). Random forest regression revealed that theory-derived cognitive variables, particularly negative appraisal of symptoms and maladaptive cognitive coping, were considered the most important predictors of PTSD symptoms post-burn.

**Conclusion**: The prevalence of probable PTSD and MDD were relatively higher in burn survivors. Theory-derived cognitive variables substantially improve predictions for PTSD symptoms post-burn.

## Abstract (es)

**Antecedentes**: El Trastorno de Estrés Post-traumático (TEPT) y los síntomas depresivos son relativamente frecuentes entre sobrevivientes a quemaduras. Varios factores de riesgo previamente reportados (por ej., severidad de la quemadura) no han predicho sistemáticamente el ajuste psicológico post-quemadura. Factores de riesgo de TEPT empíricamente derivados de meta-análisis y variables cognitivas derivadas de la teoría pueden tener gran valor predictivo.

**Objetivo:** Este estudio investigó la prevalencia de probable TEPT y depresión mayor (DM), según DSM-5, y los predictores de TEPT y síntomas depresivos en sobrevivientes de quemaduras de la explosión en el Formosa Coast Water Park del año 2015. Tres grupos de predictores fueron examinados: (a) variables relacionadas con la quemadura; (b) factores de riesgo empíricamente derivados de meta-análisis; y (c) variables cognitivas derivadas de la teoría.

**Método:** Participaron 116 sobrevivientes de quemaduras de la explosión en el Formosa Coast Water Park. La edad promedio al momento del desastre fue 22.3±4.2 años; la superficie corporal quemada total promedio fue 49.5%.

**Resultados:** De nuestros participantes, 12.9% y 20.7% cumplieron criterios para probable TEPT y DM según DSM-5 tras dos años de ocurrida la explosión en el Formosa Coast Water Park. No se observaron diferencias por género. Respecto a la predicción de síntomas de TEPT post-quemadura, las variables cognitivas derivadas de la teoría (R2 ajustado = 0.562, IC 95% [0.427, 638]) rindieron mejor y entregaron una predicción significativamente mejor que los factores de riesgo derivados de meta-análisis (R2 ajustado = 0.337, IC 95% [0.180, 0.412] y que las variables relacionadas con la quemadura (R2 ajustado = 0.313, IC 95% [0.156, 0.389]). En contraste, los tres grupos de variables examinadas entregaron similar predicción para síntomas depresivos post-quemadura (R2 ajustado = 0.267-0.295). La regresión de bosques aleatorios reveló que las variables derivadas de la teoría, particularmente valoración negativa de síntomas y afrontamiento cognitivo desadaptativo, fueron consideradas los más importantes predictores de síntomas de TEPT post-quemadura.

**Conclusión:** La prevalencia de probable TEPT y DM fue relativamente alta entre los sobrevivientes de quemaduras. Las variables cognitivas derivadas de la teoría mejoran sustancialmente las predicciones para síntomas de TEPT post-quemadura.

## Abstract (zh)

**背景**：創傷後壓力症（PTSD）與憂鬱症狀在燒傷倖存者中相對常見。數項以往報告的風險因子（例如燒傷嚴重度）對燒傷心理適應的預測不一致，源自整合分析的實徵衍生PTSD風險因子和理論衍生認知變項，可能甚具預測價值。

**目的**：本研究探討2015臺灣八仙樂園爆炸事故的燒傷倖存者中，DSM-5可能 PTSD與重鬱症（MDD）的盛行率和燒傷後PTSD與憂鬱症狀的預測因子，並對三組預測因子進行檢驗，包括：(a) 燒傷相關變項；(b) 源自整合分析的實徵衍生風險因子；以及(c) 理論衍生認知變項。

**方法**：參與者為116名臺灣八仙爆炸事故的燒傷倖存者，平均22.3±4.2歲，平均燒傷體表面積（TBSA）為49.5％。

**結果**：在八仙爆炸事故兩年後，12.9%和20.7%的參與者符合DSM-5可能PTSD和MDD，無顯著性別差異存在。在燒傷後PTSD症狀的預測上，以理論衍生認知變項的表現最佳（調整後R^2^ = .562, 95％CI [.423, .638]），且其預測效果顯著優於源自整合分析的實徵衍生風險因子（調整後R^2^ = .337, 95％CI [.180, .412]）和燒傷相關變項（調整後R^2^ = .313, 95％CI [.156, .389]）。相較前者，以上三組預測因子對燒傷後憂鬱症狀的預測效力相當（調整後R^2^ = .267-.295）。進一步採用隨機森林回歸（random forest regression）分析顯示，理論衍生認知變項—尤其症狀的負向評估和不良認知因應，是燒傷後PTSD症狀的最重要預測因子。

**結論**：在燒傷倖存者中，可能PTSD與MDD的盛行率相對較高，而理論衍生認知變項可大幅改善對燒傷後PTSD症狀的預測效果。

## 1. Introduction

The Formosa Fun Coast Water Park explosion was a massive man-made disaster in Taiwan. On 27 June 2015, thousands of people attended a ‘Colour Play Asia’ party held at the Formosa Fun Coast, a recreational water park in New Taipei City. The party organizers deployed coloured cornstarch powder, which is flammable, in the festivities. During the night, the firestorm erupted when a cloud of coloured powder ignited after being discharged from the main stage onto a large crowd. The serious fire resulted in 498 survivors of second- to third-degree burns, of which 72.9% (*n* = 363) had severe burns (i.e. total body surface area [TBSA] burned > 20%). All survivors were immediately taken to burn centres or hospitals able to cope with such problems across Taiwan. Fifteen died in the following weeks and months (Taiwan Ministry of Health and Welfare, 2015).

Events resulting in burn injuries (e.g. fires, explosions) are recognized as a potentially traumatic experience (PTEs) resulting in trauma-related psychological difficulties, such as posttraumatic stress disorder (PTSD) and major depressive disorder (MDD) (e.g. Ter Smitten, De Graaf, & Van Loey, 2011). The incidents that cause burns almost occur suddenly and can pose a considerable threat to life, which is proportional to the extent of burn injury. The severity of burn injury is determined by the % total body surface area (TBSA) burned and the depth of the burn. Severe burn injuries often lead to devastating physical consequences, such as scarring, disfigurement, pain and itching (Askay & Patterson, 2010) as well as negative psychological complications. These conditions require long-term physical rehabilitation and can significantly interfere with psychosocial functioning (Attoe & Pounds-Cornish, 2015).

PTSD and MDD are common psychological conditions following trauma. Based on the latest *Diagnostic and Statistical Manual of Mental Disorders, 5th edition* (DSM-5; American Psychiatric Association, 2013), PTSD now consists of four symptom clusters: intrusion, active avoidance, negative alterations in cognitions and mood, and alterations in arousal and reactivity. Although most individuals experience psychological distress subsequent to trauma, only a substantial minority go on to develop PTSD, with the cross-national lifetime prevalence being 3.9% in the total sample and 5.6% among the trauma-exposed (Koenen et al., 2017). PTSD and MDD are highly comorbid, as shown by a recent meta-analysis that 52% of individuals with current PTSD had co-occurring MDD (Rytwinski, Scur, Feeny, & Youngstrom, 2013).

Burn survivors exhibit higher rates of PTSD than the general population. Recent studies showed that prevalence of PTSD post-burn is 7–45%, depending on the time frame and methodology used (e.g. Lu, Lin, Chou, & Tung, 2007; Palmu, Suominen, Vuola, & Isometsä, 2011; Ter Smitten et al., 2011). The course of PTSD is likely to be chronic during the first two years post-burn. McKibben, Bresnick, Askay, and Fauerbach (2008) found that prevalence of probable DSM-IV PTSD was 35.1, 33.3, 28.6 and 25.4% at 1, 6, 12 and 24 months post-burn, with the PTSD persisting for 70% of probable cases. MDD is also common in burn survivors. Studies demonstrated that prevalence of post-burn is 10–16%, depending on the time frame and methodology (e.g. Logsetty et al., 2016; Palmu et al., 2011).

The findings that most burn survivors do not develop PTSD and MDD suggest that risk factors other than burn exposure account for their development after burn injury. In the field of burn injury, the most commonly studied risk factors include demographic characteristics, burn characteristics, premorbid psychopathology, personality factors and coping variables (Askay & Patterson, 2010; Attoe & Pounds-Cornish, 2015); however, the results have been equivocal. Regarding burn severity, some reported a significant association between %TBSA burned and PTSD symptoms (e.g. Palmu et al., 2011), whereas others failed to find a relationship (e.g. Lu et al., 2007; Wallis et al., 2006). Also, studies did not support associations between demographic variables and psychopathology post-burn (Attoe & Pounds-Cornish, 2015). Accordingly, burn severity and demographic characteristics may be unreliable for identifying burn survivors at risk for PTSD. In contrast, personality and coping variables (e.g. neuroticism, avoidance coping) have been more consistently shown to predict PTSD symptoms post-burn (Attoe & Pounds-Cornish, 2015; Sveen, Ekselius, Gerdin, & Willebrand, 2011).

In the field of psychotraumatology, a range of pre-, peri- and post-trauma risk factors have been identified for PTSD and its symptoms in trauma-exposed adults over the past decades. These findings were summarized in two comprehensive meta-analyses (Brewin, Andrews, & Valentine, 2000; Ozer, Best, Lipsey, & Weiss, 2003), which concluded that peri- and post-trauma factors have stronger predictive power of PTSD than pre-trauma factors. Specifically, Brewin et al. (2000) investigated 14 risk factors for PTSD, among which the three peri- and post-trauma factors confer the strongest risk of PTSD, i.e. trauma severity, lack of social support and post-trauma life stress, (*rs *= .23, .40 and .32). In contrast, Ozer et al. (2003) investigated seven non-demographic predictors for PTSD symptoms or diagnosis, with the focus on two types of variables: (a) person characteristics salient for psychological functioning and (b) aspects of the trauma or its sequelae. Of these seven predictors, peritraumatic dissociation yielded the largest effect size (*r* = .35), followed by social support, peritraumatic emotions and perceived life threat (*rs* = -.28, .26 and .26). Across both meta-analyses, some pre-trauma factors, such as prior trauma and prior psychological adjustment (particularly prior depression), yielded small but significant effect sizes (*rs* = .11 to .17).

In contrast to empirically-derived risk factors from the meta-analysis, theory-derived cognitive variables have received increasing attention in explaining PTSD or its symptoms, given the popularity of cognitive approaches to PTSD. According to Janoff-Bulman’s (1992) assumptive world theory, trauma deeply shatters one’s fundamental assumptions or core beliefs (e.g. the world is benevolent). Challenges to core beliefs could lead to the development of PTSD symptoms (e.g. Zhou, Wu, Fu, & An, 2015). Another influential theory is Ehlers and Clark (2000) cognitive model of PTSD, which postulates that excessively negative appraisals, trauma memory disorganization and maladaptive cognitive coping (e.g. rumination and thought suppression) are key maintaining factors for PTSD. Studies have consistently demonstrated their associations with PTSD symptoms across traumas, including assault and motor vehicle accidents (Ehring, Ehlers, & Glucksman, 2008; Kleim, Ehlers, & Glucksman, 2007). Their combined predictive power on PTSD and its symptoms were superior to that of the predictors from Ozer et al.’s meta-analysis (Ehring et al., 2008; Kleim et al., 2007).

To our knowledge, there is lack of studies evaluating the relationship of theory-derived cognitive variables and empirically-derived risk factors from the meta-analysis to posttraumatic symptomatology post-burn. These variables may be of great importance in explaining PTSD and depressive symptoms after burn injuries. Also, no studies have compared the predictive values of burn-related variables with those predictors for burn-related PTSD and depressive symptoms. Moreover, given the release of the new criteria for PTSD in DSM-5, it would be desirable to estimate the prevalence of probable DSM-5 PTSD in burn survivors.

This study aimed to address these gaps by investigating the predictive values of three sets of predictors for PTSD and depressive symptoms post-burn in burn survivors of the 2015 Formosa Fun Coast Water Park explosion two years after the incident. Based on this review, the three sets of predictors examined were: (a) *burn-related variables*, including age burned, %TBSA burned, length of hospital stay, perceived scar severity, burn-related pain and itch, and burn-related disabilities; (b) *empirically-derived risk factors from the meta-analysis*, including four peri- and post-trauma factors (i.e. perceived life threat, peritraumatic emotions, peritraumatic dissociation and perceived social support) and two pre-trauma factors (i.e. prior trauma and prior psychological adjustment), given the exploratory nature of this study; and (c) *theory-derived cognitive variables*, including core belief challenge (i.e. challenges to one’s long-standing, pervasive beliefs about the world and self) and three factors derived from Ehlers and Clark (2000) cognitive model (i.e. negative appraisals of symptoms, trauma memory disorganization and maladaptive cognitive coping). We expected that these all three sets of variables predict PTSD and depressive symptoms post-burn. Moreover, we sought to replicate the findings that theory-derived cognitive variables predict PTSD symptoms better than the factors derived from the meta-analysis (Ehring et al., 2008; Kleim et al., 2007).

## 2. Method

### 2.1. Participants and procedure

Participants were 116 burn survivors of the 2015 Formosa Fun Coast Water Park explosion. As described above, the incident resulted in 498 burn survivors, of which 15 died in the following weeks or months. The remaining 483 survivors had an average TBSA of 44.0% and mean age of 23.0 (*SD* = 4.5) years; 50.3% were female (Taiwan Ministry of Health and Welfare, 2015). Study recruitment took place between March 2016 and September 2017 through referral from physicians and self-referral from website advertisements. In all, 116 survivors participated and completed a survey approximately two years after the incident, representing 24% of total survivors at the time. The average time elapsed between the incident and the two-year survey was 24.6 months (*SD* = 0.98). The mean age of the participants at the accident was 22.3 years (*SD *= 4.2, range = 15–38) and 62.9% were female. The mean years of education was 14.8 (*SD* = 2.2). The mean %TBSA burned was 49.5% (*SD *= 19.6, range = 1 to 92), of which 75.0% having a TBSA greater than 40%. Other demographic and clinical characteristics of the sample are presented in Table 1. The Institutional Review Board of Chang Gung Medical Foundation, Taiwan, approved the study protocol. All participants signed an informed consent before participation.

**Table 1.** Demographic and clinical characteristics of the participants (*n* = 116).

Variable	%	*n*
Gender (female)	62.9	73
Relationship (unmarried)	94.8	100
Employment status		
Part-time/full-time job	35.3	41
Unemployed	31.0	36
At school/studying	33.6	39
Current/highest education		
High school/IVE, % (*n*)	30.2	35
BSc, % (*n*)	67.2	78
MSc/PhD, % (*n*)	2.6	3
	*M*	*SD*
Age (years)	24.03	4.25
Age at injury (years)	22.28	4.15
% TBSA burned	49.49	19.60
Length of hospitalization (days)	84.43	49.67
Time since burn (months)	24.60	0.97
PTSD symptoms (PDS-5)	18.39	13.41
Depressive symptoms (PHQ-9)	7.91	6.09

IVE = intermediate vocational education; BSc = Bachelor level education; MSc = Master level education; TBSA = total body surface area; PTSD = posttraumatic stress disorder; PDS-5 = Posttraumatic Diagnostic Scale for DSM-5; PHQ-9 = Patient Health Questionnaire-9.

### 2.2 Measures

#### 2.2.1. Outcome measures

##### 2.2.1.1. PTSD symptoms

The Posttraumatic Diagnostic Scale for DSM-5 (PDS-5; Foa et al., 2016) is a self-report measure assessing PTSD symptoms over the past month according to DSM-5 criteria. Twenty items assess symptoms corresponding to those in the four DSM-5 clusters: intrusion (Item 1–5), avoidance (Item 6–7), negative alterations in cognitions and mood (Item 8–14) and alterations in arousal and reactivity (Item 15–20). An additional two questions (Item 21–22) assess distress and interference caused by PTSD symptoms. Items are rated on a 5-point scale of frequency and severity (0 = not at all; 4 = six or more times a week/severe). The PDS-5 demonstrated excellent reliability (α = .95 for internal consistency, *r *= .90 for one-week test-retest reliability) as well as good diagnostic agreement with the Clinician-Administered PTSD Scale for DSM-5 (CAPS-5; Weathers et al., 2013). The optimal cut-off score for identifying probable PTSD diagnosis is 28, with sensitivity 79% and specificity 78% (Foa et al., 2016). Cronbach’s alpha of the total PDS-5 in this study was .93.

##### 2.2.1.2. Depressive symptoms

The Patient Health Questionnaire-9 (PHQ-9; Spitzer, Kroenke, Williams, & Group, 1999) is a 9-item self-report measure assessing depressive symptoms over the past two weeks based on DSM-IV and DSM-5 criteria of MDD. Items are rated on a 4-point scale (0 = not at all, 3 = nearly every day) on the basis of how much a symptom has bothered respondents. The PHQ-9 shows satisfactory reliability (α = .86–.89 for internal consistency, *r *= .84 for two-day test-retest reliability). The optimal cut-off score for identifying probable MDD diagnosis is 10, with sensitivity 88% and specificity 88% (Kroenke, Spitzer, & Williams, 2001). Cronbach’s alpha in this study was .92.

### 2.2.2. Burn-related variables

#### 2.2.2.1. Burn characteristics

Severity of burn injury (%TBSA burned), locus of burn, hospital length of hospital stay (in days) and age at time of burn.

#### 2.2.2.2. Perceived scar severity

Three items designed by Lawrence, Fauerbach, and Thombs (2006) were used: (a) subjective rating of appearance: ‘*Rate the degree of burn scarring has changed your overall appearance*’ and ‘*Rate the degree of burn scarring has changed the appearance of your face*’ both on a 6-point scale (1 = no change; 6 = very severe change); and (b) subjective rating of scar visibility: ‘*When you are in public, how often are your burn scars visible to others?*’ on a 5-point scale (1–5; 1 = none of the time; 5 = all the time). Items were summed to create a total severity score. Cronbach’s alpha in this study was .64.

#### 2.2.2.3. Burn-related pain and itch

We designed two items to assess ongoing burn-related pain and itch: ‘*what is the level of burn-related pain you experienced now?’* and *‘what is the level of burn-related itch you experienced now?*’ Items were rated on an 11-point scale (0 = none; 10 = most severe). Cronbach’s alpha in this study was .68.

#### 2.2.2.4 Burn-related disabilities

The Sheehan Disability Scale (Sheehan, Harnett-Sheehan, & Raj, 1996) is a 3-item self-report measure for assessing functional impairment in three inter-related domains: work/school, social and family life. Items are rated on an 11-point scale (0 = not at all; 10 = extremely). Cronbach’s alpha in this study was .88.

### 2.2.3. Empirically-derived risk factors

#### 2.2.3.1. Prior trauma

The trauma screen part of the PDS-5 (Foa et al., 2016) was used to assess prior trauma, including eight PTEs (e.g. disaster, accident, sexual assault). The number of PTEs endorsed was summed to create an index of prior trauma.

#### 2.2.3.2. Prior psychological adjustment

Prior depression was measured here given its close association with PTSD symptoms (Ozer et al., 2003). Three dichotomous items (Yes = 1, No = 0) originally designed by Rost, Burnam, and Smith (1993) were revised to assess the presence of prior depression before the incident (Formosa Fun Coast Water Park explosion): (1) ‘*In the past year before the incident have you had two weeks or more during which you felt sad, blue, or depressed; or when you lost all interest or pleasure in things that you usually cared about or enjoyed?*’; (2) ‘*Before the incident, have you had two years or more in your life when you felt depressed or sad most days, even if you felt okay sometimes?*’; and (3) ‘*Have you felt depressed or sad much of the time in the past year before the incident*?’. Items were summed to create a screen index. Sensitivity was 83–94% and specificity was over 90% relative to a diagnostic instrument (Rost et al., 1993).

#### 2.2.3.3. Perceived life threat

One item ‘*I felt my life was being threatened*’ was used to evaluate perceived life threat during burn accident on a 5-point scale (0 = not at all; 4 = very strongly).

#### 2.2.3.4. Peritraumatic emotions

We constructed four items to assess peritraumatic emotions. Respondents rated the extent to which they had experienced the following emotions during the accident on a 5-point scale (0 = not at all; 4 = very strongly): (a) fear, helplessness and horror; (b) anger; (c) shame; and (d) guilt. Cronbach’s alpha in this study was .75.

#### 2.2.3.5. Peritraumatic dissociation

The State Dissociation Questionnaire (SDQ; Murray, Ehlers, & Mayou, 2002) is a 9-item self-report measure that assesses different aspects of dissociation during trauma. Items are rated on a 5-point scale (0 = not at all; 4 = very strongly). The SDQ demonstrated good reliability and validity (Halligan, Michael, Clark, & Ehlers, 2003; Murray et al., 2002). Cronbach’s alpha in this study was .92.

#### 2.2.3.6 Perceived social support

The Crisis Support Scale (CSS; Joseph, Andrews, Williams, & Yule, 1992) is a 6-item self-report measure that assesses perceived social support following trauma. Items are rated on a 7-point scale (1 = never; 7 = always). The CSS demonstrated good reliability and validity (Joseph et al., 1992). Cronbach’s alpha in this study was .79.

### 2.2.4. Theoretically-derived cognitive predictors

#### 2.2.4.1. Core belief challenge

The Belief Violation Questionnaire (BVQ) is a 7-item self-report measure that assesses challenge to core beliefs following trauma. Based on Janoff-Bulman’s (1992) assumptive world theory, seven items were constructed to assess whether the following beliefs were challenged: (1) people are good and trustworthy; (2) the world is a good and safe place; (3) the world is controllable; (4) the world is fair and just; (5) I am in control of my life; (6) I am worthy; and (7) people’s behaviour is comprehensible and predictable. Items are rated on a 4-point scale (1 = not at all; 4 = very much). The BVQ exhibited adequate reliability and concurrent validity. Cronbach’s alpha in this study was .87.

#### 2.2.4.2. Negative appraisals of PTSD symptom

The Negative Appraisals of Intrusion subscale of the Response to Intrusion Questionnaire (RIQ; Clohessy & Ehlers, 1999; Murray et al., 2002) is a 6-item self-report measure that assesses negative appraisal of trauma intrusion. Items are rated on a 7-point scale (1 = totally disagree; 7 = totally agree). This subscale showed good reliability and predictive validity (Clohessy & Ehlers, 1999). Cronbach’s alpha in this study was .93.

#### 2.2.4.3. Maladaptive cognitive coping (i.e. trauma-related rumination and thought suppression)

The RIQ-Rumination subscale (Clohessy & Ehlers, 1999; Murray et al., 2002) is an 8-item self-report measure that assesses how often respondents were preoccupied with trauma and its consequences. The RIQ-Thought suppression subscale is a 6-item self-report measure that assesses how often respondents suppressed or pushed away the intrusive memories. Items are rated on a 5-point scale (0 = never; 4 = always). Both subscales demonstrated good reliability and predictive validity. Cronbach’s alpha in this study were .90 and .93, respectively. Analyses showed that both subscales were highly correlated (*r* = .66, *p* < .001). We thus standardized scores for each subscale and averaged them to create an index of maladaptive cognitive coping.

#### 2.2.4.4. Trauma memory disorganization

The Trauma Memory Questionnaire (TMQ; Halligan et al., 2003) is a 5-item self-report measure that assesses trauma memory disorganization (e.g. deficits in intentional recall). Items are rated on a 5-point scale (0 = not at all; 4 = very strongly). The TMQ showed good reliability and validity (Halligan et al., 2003). Cronbach’s alpha in this study was .90.

## 2.3. Data analysis

Data analyses were performed using SPSS18.0 and R version 3.5.0; there were no missing data. First, we conducted multiple regression analyses to examine the predictive values of three different sets of predictors for PTSD and depressive symptoms post-burn, including (a) burn-related variables (six variables); (b) empirically-derived risk factors from the meta-analysis (six variables); and (c) theory-derived cognitive variable (four variables). To compare the explained variance of the three regression models, the adjusted *R* ^2^ values (instead of *R* ^2^) were calculated because they consider the number of predictor variables included in the model. We used confidence intervals (CI) to test for significant differences in adjusted *R* ^2^ among three variables sets, following the guidelines for inferential interpretation of the overlap of CIs (Cumming, 2013). Specifically, when comparing two independent means, *p* ≤ .05 when the overlap of the 95% CI is no more than about half the average margin of error and *p* ≤ .01 when the two CIs do not overlap. The guidelines help establish the presence of significant group differences (*p* ≤ .05 to *p* ≤ .01) despite overlapping CIs. Squared semipartial correlation coefficients (*sr* ^2^) were used to represent the effect size for each regression predictor (Kelley & Maxwell, 2010). Tolerance and variance inflation factor (VIF) were calculated to assess multicollinearity.

Furthermore, random forest regression analyses were conducted using the randomForest package 4.6 in R (Liaw & Wiener, 2002) to assess the relative importance of all predictor variables of PTSD and depressive symptoms post-burn. Random forest regression is a modern non-parametric regression approach based on bootstrapping approach. This approach is highly efficient in handling nonlinearities and complex interactions among predictor variables as well as minimizing multicollinearity, as compared to conventional linear regression. In random forest regression, the most widely used index of variable importance is the percentage of increase in mean square error (%IncMSE) following the permutation of a given predictor variable. Higher %IncMSE indicates greater variable importance (Genuer, Poggi, & Tuleau-Malot, 2010).

## 3. Results

### 3.1. Descriptive statistics and bivariate correlations

Table 2 presents the means, standard deviations and zero-order correlations of all study variables. Correlations between the predictors of interest and PTSD symptoms were substantial.

**Table 2.** Means, *SD* and zero-order correlations for all study variables (*n* = 116).

Variable	1	2	3	4	5	6	7	8	9	10	11	12	13	14	15	16	17	18	19
1. Gender	—																		
2. Age burned	.16	—																	
3. %TBSA burned	−.12	−.15	—																
4. Length of stay	−.19*	−.09	.56***	—															
5. Perceived scar severity	−.19*	−.05	.64***	.52***	—														
6. Burn-related pain and itch	−.06	.03	.30**	.21*	.38***	—													
7. Burn-related disabilities	−.03	−.06	.36***	.37***	.60***	.63***	—												
8. Prior trauma	.10	.08	.27**	.26**	.28**	.25**	.16	—											
9. Prior depression	−.12	−.01	−.01	.13	.02	.16	.14	.11	—										
10. Perceived life threat	.16	−.01	.14	.03	.12	.13	.19*	.08	.01	—									
11. Peritraumatic emotions	.18	−.01	.21*	.20	.22*	.20*	.39***	.17	.17	.51***	—								
12. Peritraumatic dissociation	−.07	.05	.04	.13	−.04	.09	.25**	−.03	.13	.34***	.46***	—							
13. Perceived social support	−.11	−.08	.07	−.01	.02	−.08	−.19*	−.08	−.20	.06	−.01	.01	—						
14. Core belief challenge	−.27**	−.06	.13	.13	.09	.22*	.33***	−.08	.19*	.18	.26**	.52***	.02	—					
15. Negative appraisals of symptoms	−.10	−.04	.17	.23*	.25**	.37***	.50***	.20*	.27*	.21*	.38***	.41***	−.18	.46***	—				
16. Maladaptive cognitive coping	−.11	.03	.04	.13	.12	.35***	.41***	.11	.10	.20*	.27**	.41***	−.09	.44***	.56***	—			
17. Disorganized memory	.07	−.01	.11	.15	.05	.40***	.40***	−.10	.12	.23*	.32***	.36***	−.16	.35***	.37***	.40***	—		
18. PTSD symptoms	−.02	.06	.14	.20*	.23*	.45***	.55***	.36***	.27**	.17	.34***	.36***	−.30**	.41***	.68***	.66***	.35***	—	
19. Depressive symptoms	−.08	.14	.16	.12	.22*	.43***	.52***	.27***	.39***	.03	.22*	.18	−.35***	.34***	.54***	.42***	.30**	.75***	—
*M*	—	22.28	49.49	84.43	9.98	7.73	14.53	0.50	0.81	3.16	8.03	23.54	29.98	18.18	21.34	0.00	5.17	18.39	7.91
*SD*	—	4.15	19.60	49.67	2.79	4.02	8.58	0.82	1.15	1.25	4.50	9.93	6.42	5.08	9.31	0.91	4.46	13.41	6.09

Gender was coded as 0 for female and 1 for male. Maladaptive cognitive coping was calculated as the mean of the standardized scores of the Rumination subscale and Thought Suppression scale of the Response to Intrusion Questionnaire. TBSA = total body surface area; PTSD = posttraumatic stress disorder

*p < .05; **p < .01; ***p < .01.

### 3.2. Prevalence of probable PTSD and MDD post-burn

We estimated the prevalence of probable PTSD and MDD based on DSM-5 diagnostic algorithm. The PDS-5 and PHQ-9 were used to determine PTSD and MDD diagnosis, respectively. To meet criteria for probable DSM-5 PTSD, respondents were required to endorse one intrusion symptom, one avoidance symptom, two cognition and mood symptoms, and two arousal symptoms (Criterion B-E) on the PDS-5, as well as the presence of clinically significant distress or interference with functioning (Criterion G; operationalized as a score of 2 or higher on either Item 21 or 22). The severity threshold for symptom presence was set as a score of at least two (i.e. 2–3 times a week/somewhat) on a 5-point scale (0–4), which correspond to the conventional algorithm of the Posttraumatic Stress Disorder Checklist for DSM‐5 (PCL-5). To meet criteria for probable DSM-5 MDD, respondents were required to endorse at least one of the first two items, either anhedonia or depressed mood, as well as at least five total symptoms on the PHQ-9. A score of at least two (i.e. more than half the days) on a 4-point scale (0–3) is sufficient to qualify as a symptom, with the exception of the suicidal ideation item (counted as a symptom if scored 1 or greater) (Manea, Gilbody, & McMillan, 2015).

Two years after the Formosa Fun Coast Water Park explosion, 12.9% (*n* = 15) of participants met criteria for DSM-5 PTSD and 20.7% (*n* = 24) met criteria for DSM-5 MDD. No gender differences were observed in the prevalence of PTSD (female: 11.0%, male: 16.3%; *χ^2^*(1) = 0.68, *p *= .409) and MDD (female: 24.7%, male: 14.0%; *χ^2^*(1) = 1.89, *p *= .169). Nine of the 15 participants (60%) with probable PTSD had comorbid MDD. As a comparison, predefined cut-off scores for the PDS (i.e. 28; Foa et al., 2016) and PHQ-9 (i.e. 10; Kroenke et al., 2001) were also used to estimate the prevalence of PTSD and MDD. This procedure resulted in steep increase of a probable PTSD rate to 22.4% (*n* = 26) and a probable MDD rate to 36.2% (*n* = 42).

### 3.3. Predictors of PTSD and depressive symptoms post-burn

Multiple regression analyses examined the predictors of PTSD and depressive symptoms post-burn. The predictive power of three sets of variables were examined separately: (a) *burn-related variables*: age burned, %TBSA burned, length of stay, perceived scar severity, burn-related pain and itch, and burn-related disabilities; (b) *empirically-derived risk factors from the meta-analysis*: prior trauma, prior depression, perceived life-threat, peritraumatic negative emotion, peritraumatic dissociation and perceived social support; and (c) *theory-derived cognitive variables*: core belief challenge, negative appraisals of symptoms, trauma memory disorganization and maladaptive cognitive coping. As shown in Table 3, three sets of variables all strongly predicted PTSD and depressive symptoms post-burn. For the prediction of PTSD symptoms post-burn, the non-overlapping CI for theory-derived cognitive variables (adjusted *R* ^2^ = .562, 95% CI [.423, .638]) as compared to the CI for empirically-derived risk factors from the meta-analyses (adjusted *R* ^2^ = .337, 95% CI [.180, .412]) and burn-related variables (adjusted *R* ^2^ = .313, 95% CI [.156, .389]) indicates that theory-derived cognitive variables had significantly greater predictive power for PTSD symptoms than the other two variables sets (*p *< .01). For the prediction of depressive symptoms post-burn, no significant differences were noted across variable sets in predictive power (adjusted *R* ^2^ = .295, .267, .295). Regarding multicollinearity, tolerance (range: .41–.96) and VIF (range: 1.04–2.42) for all predictors in each model were within acceptable limits.

**Table 3.** Multiple regression predicting PTSD and depressive symptoms post-burn by different set of variables (*n* = 116).

Variable	PTSD symptoms			Depressive symptoms			VIF	Tolerance
	*β*	*p*	*sr*^2^	*β*	*p*	*sr*^2^		
**Burn-related variables**								
Age burned	.08	.289	.007	.17	.040	.027	1.04	.96
%TBSA burned	−.03	.760	.001	.10	.390	.005	2.02	.50
Length of stay	.09	.350	.005	−.05	.628	.001	1.58	.63
Perceived scar severity	−.18	.144	.013	−.19	.131	.014	2.42	.41
Burn-related pain and itch	.18	.088	.018	.14	.181	.011	1.73	.58
Burn-related disabilities	.53	.000	.121	.54	.000	.125	2.31	.43
*R*^2^	.349			.332				
Adjusted *R*^2^,95% CI	.313 [.156, .389]			.295 [.115, .392]				
**Empirically-derived risk factors from the meta-analysis**								
Prior trauma	.32	.000	.094	.21	.012	.041	1.06	.94
Prior depression	.12	.137	.013	.28	.001	.071	1.10	.91
Perceived life threat	−.01	.944	.000	−.07	.434	.004	1.40	.72
Peritraumatic emotions	.13	.199	.010	.12	.246	.009	1.63	.61
Peritraumatic dissociation	.30	.001	.069	.12	.203	.010	1.32	.76
Perceived social support	−.25	.002	.060	−.27	.001	.069	1.05	.95
*R*^2^	.372			.305				
Adjusted *R*^2^, 95% CI	.337 [.180, .412]			.267 [.088, .365]				
**Theory-derived cognitive variables**								
Core belief challenge	.02	.776	.001	.07	.439	.004	1.39	.72
Negative appraisal of symptoms	.45	.000	.122	.41	.000	.103	1.62	.62
Maladaptive cognitive coping	.39	.000	.095	.13	.185	.011	1.62	.62
Disorganized memory	.02	.767	.000	.07	.455	.003	1.27	.79
*R*^2^	.577			.320				
Adjusted *R*^2^, 95% CI	.562 [.423, .638]			.295 [.134, .402]				

Standardized regression coefficients are shown across the rows where individual variables are listed. PTSD = posttraumatic stress disorder; *sr* ^2^ *= *squared semi-partial correlation coefficient; VIF = variance inflation factor; TBSA = total body surface area.

Random forests regression investigated the relative importance of all predictors of PTSD and depressive symptoms post-burn. Table 4 presents %IncMSE values of all predictors that indicate the rank of variable importance. Regarding the prediction of PTSD symptoms post-burn, maladaptive cognitive coping and negative appraisal of symptoms were the two most important predictors (%IncMSE = 16.06 and 15.44), followed by perceived social support and burn-related disabilities (%IncMSE = 10.56 and 10.37). The remaining variables were relatively less important predictors (%IncMSE = −1.67–5.78). Regarding the prediction of depressive symptoms post-burn, prior depression, negative appraisal of symptoms, perceived social support and burn-related disabilities were the four most important predictors (%IncMSE = 12.45, 11.63, 10.88 and 10.81, respectively). Other predictors were less potent predictors (%IncMSE = −0.60–6.82).

**Table 4.** Random forest regression: relative importance of the predictors of PTSD and depressive symptoms post-burn (*n* = 116).

Variable	%Increase MSE	
	PTSD symptoms	Depressive symptoms
Gender	−1.67	0.78
Age burned	1.75	4.91
%TBSA burned	0.08	−0.60
Length of stay	1.73	1.99
Perceived scar severity	0.02	3.39
Burn-related pain and itch	5.78	5.72
Burn-related disabilities	10.37	10.81
Prior trauma	2.96	0.37
Prior depression	3.54	12.45
Perceived life threat	1.70	1.09
Peritraumatic emotions	2.34	−0.08
Peritraumatic dissociation	2.19	−0.24
Perceived social support	10.56	10.88
Core belief challenge	3.09	6.82
Negative appraisal of symptoms	15.44	11.63
Maladaptive cognitive coping	16.06	6.57
Disorganized memory	2.82	−0.49
Mean of squared residuals	83.82	21.77
%Variance Explained	53.01	40.87

Gender was coded as 0 for female and 1 for male. MSE = mean square error; PTSD = posttraumatic stress disorder; TBSA = total body surface area.

## 4. Discussion

This study found the current prevalence of probable PTSD and probable MDD based on DSM-5 criteria to be 12.9 and 20.7%, respectively, among burn survivors two years after the 2015 Formosa Fun Coast Water Park explosion in Taiwan. To our knowledge, this is the first study investigating the prevalence of probable DSM-5 PTSD among burn survivors. For the prediction of PTSD symptoms post-burn, we found that theory-derived cognitive variables (core belief challenge, negative appraisals of symptoms, trauma memory disorganization and maladaptive cognitive coping), empirically-derived factors from the meta-analysis (prior trauma, prior psychological adjustment, perceived life threat, peritraumatic emotions, peritraumatic dissociation and social support) and burn-related variables (age burned, %TBSA burned, length of hospital stay, perceived scar severity, pain and itch, and burn-related disabilities) all showed significant and substantial predictive values. Notably, theory-derived cognitive variables provided significantly better prediction than burn-related variables and empirically-derived risk factors. In contrast, the predictive values for depressive symptoms post-burn were closely similar across three sets of variables. Furthermore, maladaptive cognitive coping and negative appraisal of symptoms were the two most important predictors of PTSD symptoms post-burn, followed by perceived social support and burn-related disabilities.

In this study, the probable PTSD rate two years post-burn was 12.9%, approaching the lower limit of the previous estimates (7–45%). The probable MDD rate was 20.7%, which is higher than those reported earlier (10–16%). Notably, most previous studies have limited their investigation in the first years since the burn, and only a few studies assessed the prevalence of PTSD and MDD at more than a year post-burn (e.g. McKibben et al., 2008; Ter Smitten et al., 2011). Ter Smitten et al. (2011) found the 12-month prevalence of PTSD to be 7% 1–4 years post-burn using the diagnostic interview. McKibben et al. (2008) found the prevalence of probable PTSD to be 25.4% at two years post-burn using the PCL-5. Our finding, although slightly higher than that reported by Ter Smitten et al., is only half of the prevalence reported by McKibben et al. A possible explanation is the different algorithms used to determine PTSD. Our estimate was based on DSM diagnostic algorithm but McKibben et al.’s based on the ‘cut-off’ method. Notably, using the cut-off method (PDS-5 ≥ 28) resulted in our prevalence estimate to turn out to be 22.4%, which is close to McKibben et al.’s estimate. However, this method was not applied as it may result in overestimating the prevalence of the disorder when the prevalence is low (McDonald & Calhoun, 2010).

Both our results of multiple linear regression and random forest regression corroborate earlier findings of Kleim et al. (2007) and Ehring et al. (2008) that cognitive variables from Ehlers and Clark’s model of PTSD exerted a much stronger predictive validity than empirically-derived risk factors from the meta-analysis. Altogether, these findings lend support to the application of Ehlers and Clark’s model across trauma populations. The findings are clinically relevant as they suggest the potential risk factors for identifying people at risk for PTSD after burn. For instance, people who are inclined to negatively appraisals of their burn injury and its consequences (e.g. intrusion of injury experience) are likely to show persistent PTSD symptoms. Furthermore, although all the cognitive variables examined were associated with PTSD and depressive symptoms, only some of them showed significant prediction. One of the noteworthy results herein is that negative appraisals of symptoms predicted both PTSD and depressive symptoms, suggesting that this cognitive bias may account for comorbid PTSD and MDD.

The finding that negative appraisal of symptoms and maladaptive cognitive coping were the most important predictors of PTSD symptoms post-burn is of theoretical and practical interest. Theoretically, both variables were derived from Ehlers and Clark (2000) model. We may therefore wish to apply this theory to explain post-burn traumatic stress disorders; however, the unique circumstances surrounding burn patients (e.g. disfigurement, body image problems and stigmatization) need to be considered. The clinical implication is that cognitive variables may play a pivotal role in post-burn psychosocial adjustment. Some of the cognitive variables examined, including negative appraisals and maladaptive cognitive coping (i.e. trauma-related rumination and thought suppression), may maintain PTSD symptoms and can be targets or change mechanism of psychotherapy. Recent studies indicate that trauma-focused psychotherapy (e.g. prolonged exposure and cognitive therapy) can effectively prevent chronic PTSD in recent survivors (e.g. Shalev et al., 2012), and belief change is recognized as a key mechanism of prolonged exposure (Cooper, Clifton, & Feeny, 2017). Therefore, early use of trauma-focused psychotherapy may promote a reduction of PTSD symptoms after burn. However, burn-related physical impairment and rehabilitation challenges may impose significant barriers to participation, and interventions should be tailored to fit burn patients’ needs.

Of additional interest are the findings that three sets of variables produced closely similar predictions on depressive symptoms post-burn. Moreover, the predictive value of theory-derived cognitive variables was lower for depressive symptoms than for PTSD symptoms based on the CI estimates of adjusted *R* ^2^. These findings are somewhat expected as the cognitive predictors examined were derived from theories of PTSD. For instance, the ruminative process examined here refers to rumination about the trauma and its consequences, which is distinguished from depressive rumination proposed in Nolen-Hoeksema’s response styles theory. The latter refers to a mode of dwelling on one’s symptoms of distress and their potential causes and consequences, and has been established as a risk factor for depression given its robust association with MDD and depressive symptoms (Nolen-Hoeksema, Wisco, & Lyubomirsky, 2008). The prediction for depressive symptoms post-burn are likely be improved by incorporating variables derived from theories of depression, such as depressive rumination and dysfunctional attitudes.

Several caveats of this study need to be considered. First, because our participants suffered somewhat more severe burn injuries relative to total survivors (44.0 vs. 49.0%), the generalizability of our findings to all survivors of the Formosa Fun Coast Water Park explosion warrants careful consideration. Second, given the cross-sectional nature of data, we cannot determine the causality among the predictors examined and outcome variables. A prospective design is necessary to confirm and extend the current findings. Third, some of the predictor variables examined involved the recall of subjective experience during or prior to the incident (e.g. peritraumatic dissociation and prior depression). Thus, we cannot rule out the occurrence of recall bias that threats the validity of the self-report data. Last, formal diagnosis of PTSD and depression cannot be established using the self-report measure alone. Our findings warrant replication using a structured diagnostic interview such as the CAPS-5.

Despite these caveats, our findings may advance current knowledge concerning the development of PTSD and depressive symptoms following burn injury. This study is the first to examine the prevalence of probable DSM-5 PTSD in burn survivors. Using a sample of burn survivors of the 2015 Formosa Fun Coast Water Park explosion, we found that probable PTSD and MDD are relatively prevalent among burn survivors two years post-burn. A remarkable finding was that theory-derived cognitive variables provided much greater prediction than risk factors from the meta-analysis and burn-related variables. In conclusion, our findings highlight the importance of incorporating psychological theories of PTSD to account for post-burn traumatic stress disorders.
